# The Chemical Defensome: A Survey of Environmental Sensing and Response Genes in Copepods

**DOI:** 10.3390/ijms26041546

**Published:** 2025-02-12

**Authors:** Vittoria Roncalli, Daniela Ascione, Chiara Lauritano, Ylenia Carotenuto

**Affiliations:** 1 Integrative Marine Ecology Department, Stazione Zoologica Anton Dohrn, Villa Comunale, 80121 Napoli, Italy; daniela.ascione@szn.it (D.A.); ylenia.carotenuto@szn.it (Y.C.); 2 Ecosustainable Marine Biotechnology Department, Stazione Zoologica Anton Dohrn, Via Acton 55, 80133 Napoli, Italy

**Keywords:** detoxification, transcriptomics, environmental stress, biomarkers, zooplankton

## Abstract

Highly conserved among eukaryotes, the chemical defensome protects organisms against chemical stressors and helps to reestablish the altered homeostatic state. The defensome includes genes such as transporters (e.g., *adenosine triphosphate ATP-binding cassette*), phase I and phase II detoxification enzymes, and antioxidant enzymes. During their life cycle, planktonic copepods, the most abundant and ubiquitous metazoans on Earth, are exposed to many environmental stressors that impair their survival and fitness. Here, using high-quality publicly available transcriptomic data, defensome genes were searched in copepods belonging to different orders and living in different environments (e.g., Antarctic, Subarctic, Mediterranean). Gene expression responses were investigated in four calanoids exposed to different stresses to identify a common and species-specific detoxification system. Our results confirm that the defensome is highly conserved among copepods but also report differences in the relative contribution of genes among species living in different habitats, suggesting a fitness adaptation to environmental pressures. The genes provided here can be used as biomarkers of chemical defense and can also be tested in other planktonic organisms to assess the “health” of marine organisms, which is useful for understanding environmental adaptations and they can be used to assess changes and make predictions at the population and community levels.

## 1. Introduction

Broadly conserved across metazoans, the defensome is a collection of genes and pathways that help an organism to counteract homeostatic disruptions associated with stress. The chemical defensome includes genes that encode proteins used to sense, transform, and eliminate toxicants, serving as a protector from environmental chemicals [1,2]. Due to the diversity of chemical stressors, which can include microbial products, naturally occurring organic and inorganic compounds, and anthropogenically derived compounds, the chemical defensome includes classes of genes that can activate an orchestrated and coordinated specific or non-specific response to the stressor. After sensing a potential threat, the first line of cellular defense against amphipathic or slightly lipophilic compounds includes the activation of the *adenosine triphosphate ATP-binding cassette* (*ABC*) or multidrug efflux transporters (e.g., *p-glycoproteins and multidrug resistance proteins*) [1,2,3]. The first role of these transporters is to alter the entry of the toxicant into the cell and to impair its movement across membranes; however, if a toxicant succeeds in entering the cytoplasm, the organism depends on biotransformation, which is generally based on two phases of detoxification. The first phase of detoxification (phase I) is characterized by the activity of oxidative enzymes such as *cytochromes P450* (*CYPs*), *flavoprotein monooxygenases* (*FMOs*), and *aldehyde dehydrogenases* (*ALDHs*) that oxidize chemical compounds into more hydrophilic metabolites. In the second phase (phase II), toxicants are reduced, conjugated, or hydrolyzed by detoxification enzymes including *glutathione-S-transferases* (*GSTs*), *sulfotransferases* (*SULTs*), *UDP-glucuronosyl transferases* (*UGTs*), and *aldo-keto reductases* (*AKRs*) [1,2,3]. As a final phase, also known as phase III detoxification, efflux transporters are involved in the excretion of the transformed stressor from the organism. In addition to this defense mechanism, to maintain cellular homeostasis, an organism must inactivate and eliminate endogenous signaling molecules, such as eicosanoids and reactive oxygen species (ROS). This requires the activity of antioxidant enzymes such as *superoxide dismutases* (*SODs*), *catalases* (*CATs*), and *peroxidase*. The defensome also includes genes such as *metallothioneins* (*MTs*) or molecular chaperones (e.g., *heat shock proteins*) that are activated in response to specific stressors such as heat stress, heavy metals, or free radicals (reviewed in [4,5]).

Planktonic copepods, the most abundant and ubiquitous metazoans on Earth [6], play a vital role in marine ecosystems, serving as the key link between the lower and higher trophic levels [7,8]. Furthermore, with their diel vertical migrations or long dormant phases, they transfer energy from primary producers and the microbial loop up to higher trophic levels, significantly contributing to the biological pump (reviewed by [9]). During their lives, copepods are constantly exposed to physical, chemical, and biological challenges that impact their homeostatic state, exhibiting extreme sensitivity to the environment [10]. The increasing challenges posed by climate change and environmental pollution have highlighted the need to implement biomonitoring strategies for a better understanding of ocean health. Assessing the physiological state of copepods in natural populations, which can be accomplished by measuring the expression of specific biomarkers, can improve our ability to predict how organisms respond to environmental stressors. Furthermore, a better assessment of the copepod physiology can also be important in aquaculture for the significant role of these organisms either as parasites (“sea lice”, primarily in the Lepeophtheirus and Caligus genera infesting salmons) or as source of live feed for larvae of commercial fish species [11,12]. In the last decades, with the increase in high-quality molecular resources, there have been efforts to identify genes that could be used as potential indicators of copepods’ survival, development, growth, and fecundity. Using the calanoid *Calanus finmarchicus* (Gunnerus, 1770) as a model, several studies have characterized the diversity of genes associated with detoxification [13,14,15], signaling systems [16,17,18], photoreception [19], reproduction, and larval development [20]. However, despite its conservation among metazoans and its role in response to a variety of environmental stressors, to date little is known about the chemical defensome in copepods. In a recent study, an evolutionary analysis of three detoxification-related gene families (*ABCs*, *CYPs*, and *GSTs*) for the calanoid *Eurytemora affinis* (Poppe, 1880), the cyclopoid *Paracyclopina nana* (Smirnov, 1935), and two harpacticoids *Tigriopus japonicus* (Mori, 1938) and *T. kingsejongensis* (Park, S. Lee, Cho, Yoon, Y. Lee, and W. Lee, 2014), has shown that there are species-specific differences in their numbers of genes, which could reflect different physiological adaptations [21]. More recently, a survey on the transcriptome of *C. finmarchicus* from the Gulf of Maine has shown that the defensome genes in this copepod differ from those previously reported in *E. affinis*, in the sea urchin *Strongylocentrotus purpuratus* (Stimpson, 1857), and in the anemone *Nematostella vectensis* (Stephenson, 1935) [22]. The authors reported that except for the *ABC*, *GST*, and *AKR* classes, the number of copepod defensive genes is lower compared with the ones identified in *S. purpuratus* and *N. vectensis*, whereas the *SOD* class was over-represented in *C. finmarchicus*. Taken together, the results from both studies highlight the importance of having more genomic studies to uncover lineage-specific adaptive strategies in copepods. As a proof-of-concept, the expression of such defensive genes should be assessed in different copepod species exposed to well-defined environmental stresses.

Here, we survey high-quality publicly available (NCBI) “omic” resources for copepods (11 transcriptomes and 1 genome) belonging to different orders and living in different environments (e.g., Antarctic, Subarctic, Mediterranean) to identify a collection of genes belonging to the chemical defensome. Exploring gene expression responses in four calanoids exposed to different stresses, we provide evidence that in spite of similarities in the numbers of genes, the expression of them is species-specific and stressor-specific. To our knowledge, to date, this study is the first complete survey of copepod defensomes. Using a systematic comparative analysis, we show how gene distribution and transcriptional regulation differ between species, likely reflecting different adaptations to environmental pressures. The genes provided in this study could be used as biomarkers for environmental biomonitoring not only in copepods but also in other zooplankters.

## 2. Results

### 2.1. Identification of Transcripts Involved in the Copepod Chemical Defensome

**Figure 1:**
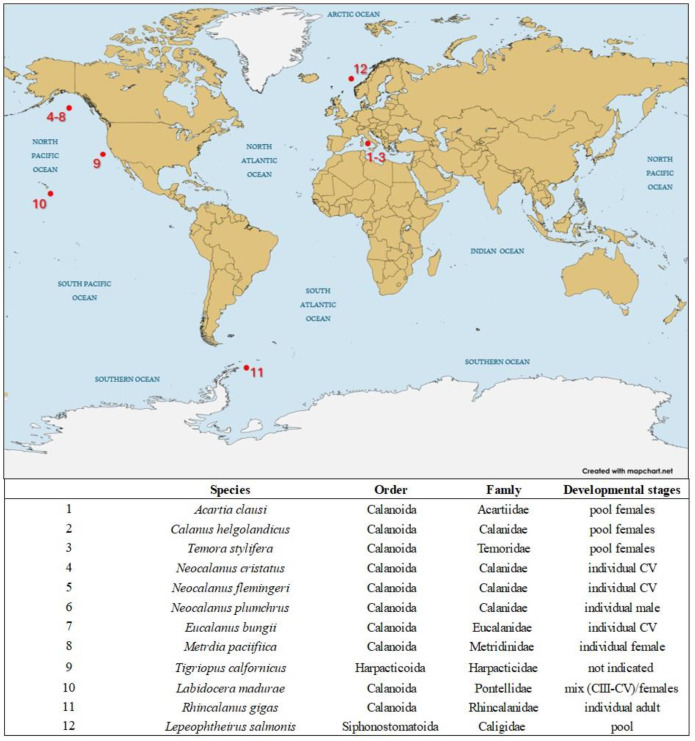
Geographical distribution of the copepods used in this study for the survey of the chemical defensome. For each copepod, species, order, family, and the developmental stage/s used for the generation of the transcriptome/genome are listed. Created with https://www.mapchart.net (accessed on 2 February 2025).

**Figure 2:**
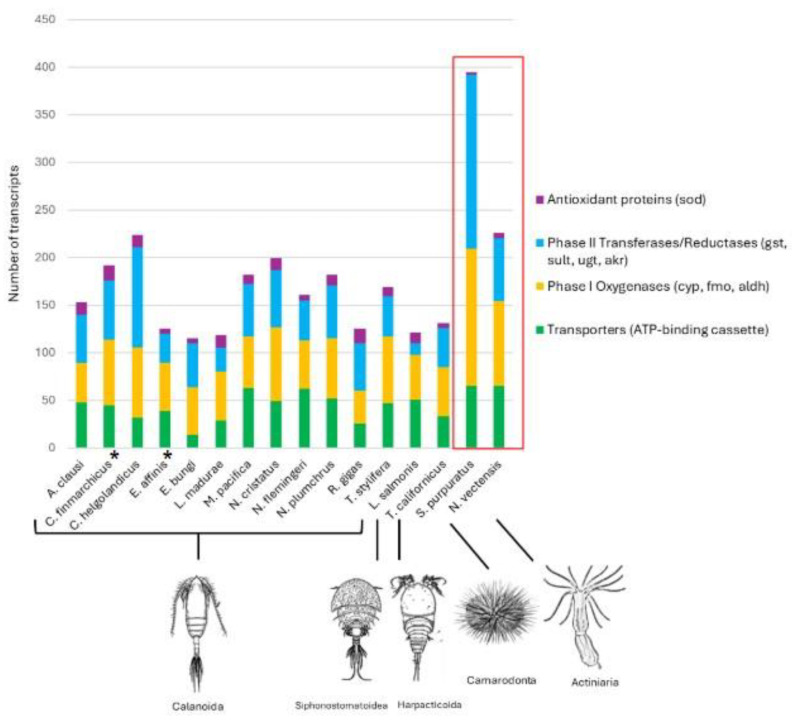
Distribution of defensome encoding transcripts in copepods (this study) compared with other copepods (*Calanus finmarchicus* and *Eurytemora affinis* (*)) [22], and the sea urchin *Strongylocentrotus purpuratus*, and the anemone *Nematostella vectensis* (red box). Genes have been divided into four classes as follows: transporters (*ATP-binding cassette*), phase I oxygenases (*ALDH*, *CYP*, *FMO*), phase II transferases/reductases (*AKR*, *GST*, *SULT*, *UGT*), and antioxidant protein (*SOD*). Copepod gene diversity is shown for the order Calanoida (in alphabetic order): *Acartia clausii* (Giesbrecht, 1892), *C. finmarchicus C. helgolandicus*, *E.affinis*, *E. bungii*, *Neocalanus cristatus* (Krøyer, 1848), *N. flemingeri*, *Neocalanus plumchrus* (Marukawa, 1921), *L. madurae*, *Metridia pacifica* (Brodskij, 1950), *R. gigas*, *T.stylifera*), Siphonostomatoida (*L. salmonis*), and Harparticoida (*T. californicus*).

An in silico workflow was used to mine eleven transcriptomic (and one genomic) resources to identify defensive genes based on their functional annotation in copepods belonging to three different orders (Calanoida, Siphonostomatoid, Harpacticoid) and living in different environments, as shown in Figure 1. All the searched components of the copepod defensome, including the *ATP-binding cassette transporters* (*ABCs*), phase I and phase II enzymes, and the antioxidant superoxide dismutases (*SODs*), were found in the 12 copepod species (Figure 1 and Figure 2). The total number of transcripts across copepods ranged from 115 in *Eucalanus bungii* (Giesbrecht, 1893) to a maximum of 224 in *Calanus helgolandicus* (Claus, 1863). The entire collection of genes was found in six copepods, while in the others there were some genes missing. *FMO* was absent in *E. bungii*, *Neocalanus flemingeri* (Miller, 1988), *Tigriopus californicus* (Baker, 1912), and *C. helgolandicus*; *SULT* was missing in *Lepeophtheirus salmonis* (Krøyer, 1837); and *UGT* was not found in *E. bungii*, *Temora stylifera* (Dana, 1853–1855), *L. salmonis*, and *N. flemingeri*. Classifying the genes by their roles, the phase I and phase II classes, which include the highest number of transcripts, show a comparable coverage across species. The transporter class, which includes only the *ABC-cassette* transcripts, is relatively very abundant (27% average) compared to the other two classes, with the highest number (n = 63) in *M. pacifica* and the lowest number (n = 14) in *E. bungi*. The distribution of the antioxidant *superoxide dismutases* (*SODs*) is somehow consistent across all copepods, representing 4% of the defensome in *E. bungii* and *T. californicus* up to 12% in *Labidocera madurae* (Scott A., 1909) and *Rhincalanus gigas* (Brady, 1883) (Figure 2).

### 2.2. Relative Expression of Defensome Genes in Calanus finmarchicus, C. helgolandicus, Temora stylifera, and Acartia clausi Exposed to Stressors

**Figure 3:**
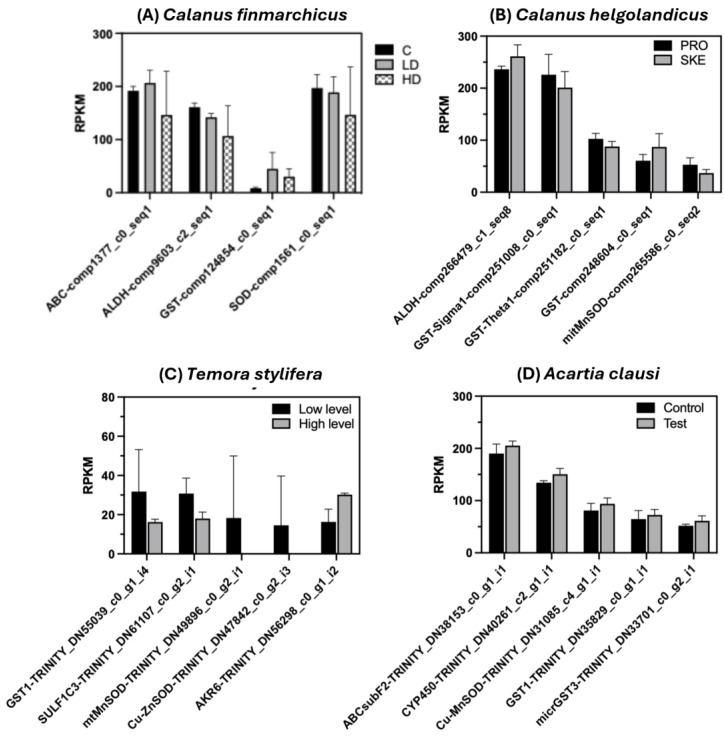
Relative expression of responsive genes identified in the chemical defensome of the copepods *Calanus finmarchicus*, *C. helgolandicus*, *Temora stylifera*, and *Acartia clausi* exposed to stressors. (**A**) Relative expression in *C. finmarchicus* females fed the toxic dinoflagellate *Alexandrium fundyense* at two doses (low [LD]) and high [HD]) and the control diet *Rhodomonas baltica* for two days. (**B**) Relative expression in *C. helgolandicus* females fed the toxic diatom *Skeletonema marinoi* (SKE) and the control diet *Prorocentrum minimum* (PRO) for 5 days. (**C**) Relative expression in *T. stylifera* females collected in the Gulf of Naples during two consecutive sampling weeks, when low (30th May) and high (23rd May) levels of harmful oxylipins were measured in the natural phytoplankton assemblage. (**D**) Relative expression in *A. clausi* females exposed for four days to elutriates of polluted sediments containing high concentrations of polycyclic aromatic hydrocarbons, PAHs, and heavy metals, compared with controls. Different names indicate different transcripts. Bars are mean standard deviation (n = 3 replicates). For all three species, relative expression is normalized by length (RPKM).

Exposure to the toxic dinoflagellate *Alexandrium fundyense* (Balech, 1985) for two days induced in adult females of *Calanus finmarchicus* significant changes in the expression of four transcripts belonging to the copepod’s defensome annotated as the *ABC transporter*, *ALDH*, *GST*, and *SOD* (Figure 3A). The relative expression for the three transcripts was > 100 RPKM (*ABC*, *ALDH*, *SOD*), while for the *GST* the average expression was below 30 RPKM. Three transcripts (*ABC*, *ALDH*, *GST*) were differentially regulated in individuals on the LD diet compared with the control diet (*p* < 0.0001 for *ALDH* and *GST*; *p* = 0.00053 for *ABC*), with *GST* and *ABC* being upregulated and *ALDH* downregulated. In the HD individuals, downregulation was observed for *ALDH* (*p* = 000234) and for *SOD* (*p* = 0.000607) (Figure 3A). At 5 days, a single *GST Omega* showed differences in expression upon feeding on the toxic algae, with a higher expression in both LD and HD compared with control individuals (data not shown; [23]).

In the congener *C. helgolandicus*, expression changes were observed in females feeding for five days on a toxic alga compared with a control diet for *ALDH*, three *GSTs*, and *SOD*. Compared with control individuals, upregulation was found in females feeding on the oxylipin producing *Skeletonema marinoi* (SKE) for *ALDH* (*p* < 0.000001) and one of the *GSTs* (*p* < 0.000001), with an average relative expression ranging between 87 and 260 RPKM (Figure 3B). In contrast, downregulation was found for the *GSTs sigma* (*p* < 0.000001) and theta (*p* = 0.0158) and the mitochondrial *MnSOD* (*p* = 0.00610), with an average expression between 88 and 201 RPKM (Figure 3B). Changes in the expression of three phase II genes, *GST*, *SULF*, and *AKR*, and two *SODs* were also reported in *T. stylifera* females collected at sea during different biotic conditions. With an average relative expression <20 RPKM, *GST1(delta)* and *SULT1* showed significant downregulation (*p* = 0.000002 and *p* = 0.00014, respectively) in individuals collected when the natural phytoplankton assemblage produced higher levels of oxylipins compared with the earlier week, when a lower concentration of cytotoxins was measured (Figure 3C). Both *SODs*, mitochondrial *MnSOD* and *Cu-ZnSOD*, were downregulated as well, with zero relative expression (*p* < 0.00001). Conversely, the *AKR* gene was significantly upregulated in *T. stylifera* females collected during the high-level compared with the low-level week (*p* = 0.000019) (Figure 3C). Lastly, in *A. clausi* females, exposure for four days to elutriates of polluted sediments containing high concentrations of PAHs and HMs, compared with controls, induced changes in expression for five transcripts encoding for the ABC transporter, phase I (*CYP*), phase II (*GSTs*), and antioxidant *SODs* (Figure 3D). All transcripts were strongly upregulated in females exposed to the elutriate, compared with those in control seawater, with *ABC* and *CYP450* having a very high relative expression (on average, ranging from 151 to 205 RPKM) (*p* < 0.000001). *GST1*, *GST3*, and the antioxidant *Cu-MnSOD* also showed a high relative expression, on average 60–94 RPKM (*p* < 0.01) (Figure 3D).

## 3. Discussion

In planktonic copepods, the constant exposure to physical, chemical, and biological challenges alters the maintenance of cellular homeostasis, which is required for a good physiological state. To counterbalance the stress associated with exposure to chemicals, including exogenous compounds (xenobiotics) and endogenously produced toxicants, organisms regulate the expression of genes belonging to the chemical defensome, a collection of genes highly conserved across various animal taxa [1,2,24,25]. As an orchestrated system, the chemical defensome includes genes responsible for regulating the entry of the xenobiotics (the *ATP-binding cassette protein* superfamily), their detoxification, and excretion. Detoxification includes phase I enzymes (the *cytochrome P450* superfamily, *flavoprotein monooxygenases*, *aldehyde dehydrogenases*) responsible for biotransformation and phase II ones, which regulate the conjugation of the toxicant (*glutathione-S-transferases*, *sulfotransferases*, *UDP-glucuronosyl transferases*, and *aldo-keto reductases*) [1,2,26]. Phase II also includes antioxidant enzymes such as *superoxide dismutases*, which are specifically activated to restore homeostasis upon antioxidative stress induced by endogenous signaling molecules such as ROS [2]. Over the years, many studies in copepods have measured the expression of genes belonging to the chemical defensome to assess the organismal physiological state in response to exposure to chemical stressors [27]. The most used biomarkers, selected based on their specific mode of action, have been antioxidant enzymes, *catalases*, *glutathione peroxidases*, *peroxiredoxins*, *cytochrome P450 oxidases*, and *glutathione S-transferases*. However, common to many studies, the gene expression results have shown complicated patterns, dependent on the duration, concentration, and the type of stressor, as well as the specific genes selected as single biomarkers [27].

The increased availability of copepod high-throughput sources (transcriptomes and genomes) has provided, in the last decades, a good opportunity to place biomarkers within a gene family, with the advantage of avoiding the a priori selection of candidate genes. To date, among marine organisms, the chemical defensome network has been described only in the sea urchin *Strongylocentrotus purpuratus* [1], the sea anemone *Nematostella vectensis* [2], and the copepods *Eurytemora affinis* and *Calanus finmarchicus* [21,22]. The first two investigated copepod defensomes have shown to possess all the representative genes and share with *S. purpuratus* and *N. vectensis* a similar percentage of transporters and phase I and phase II detoxification genes. Some differences were found in the percentage of the antioxidant (*SOD*), where the number was significantly higher in *C. finmarchicus* compared with all other organisms; differences that could be related to the different resources mined for the identification (genome vs. transcriptome) [22]. In our study, all gene representatives of the chemical defensome have been found in 50% of the copepods surveyed. We believe that the lack of representatives for some of the classes (e.g., *FMO*, *AKR*, *UGT*, and *SULT*) is due to the differences in the resources (e.g., yields, sequencing platform, assembly, developmental stage, level of annotation); however, for a full understanding of the copepod chemical defensome, more species and stages will need to be surveyed, and possibly, the transcriptomic results will need to be confirmed by future genome sequencing.

The diversity of defensome genes in copepods can be influenced by habitats or life strategies. In a recent study, an evolutionary analysis revealed that the number of orthologs of the *ABC cassette*, *CYP450*, and *GST* families was different between the calanoid *E. affinis*, the cyclopoid *Paracyclopina nana*, and the harpacticoids *Tigriopus japonicus* and *T. kingsejongensis* [21]. The authors suggest that different life strategies, with *E. affinis* pelagic and *Tigriopus* spp. Benthic, could influence the xenobiotic metabolism and explain the gene diversity found within the *GST* family [21]. In our study, we also found differences between calanoid, harpacticoid, and siphonostomatoid copepods for phase II enzymes; *L. salmonis* (siphonostomatoid) had a lower percentage of genes compared with the copepods from the other two orders. These differences were even more pronounced within the *GST* family, with calanoids reporting a high number (average n = 32) compared with the benthic harparticoid *T. californicus* (n = 22) and the parasitic siphonostomatoid *L. salmonis* (n = 10). Such lower abundance/diversity could also reflect an evolutionary strategy for resource allocation in copepod orders/species with very specialized lifestyles. While on the one hand, we confirm differences between orders, we also report high consistency in the distribution of defensome genes within the Calanoida order. This order includes 14 different copepods, with five members of the family Calanidae (*N. flemingeri*, *N. plumchrus*, *N. cristatus*, *C. finmarchicus*, and *C. helgolandicus*) having a complex life cycle characterized by a period of dormancy (a “diapause” or low metabolism in over-wintering stages) [28,29], a member of the non-calanid myelinate calanoids (the eucalanid *E. bungii*), and the amyelinate *Metridia pacifica* known for diel vertical migration and bioluminescence [30]. Taken together, we can speculate that if on the one hand different life strategies (planktonic vs. benthic habitats), feeding behaviors (ambush and/or raptorial feeders vs. feeding current feeders), and dietary preferences (herbivory, carnivory, parasitism), can influence the diversification of genes within the chemical defensome (calanoids and harparticoids being free-living, as opposed to the siphonostomatoid being parasitic), some gene conservation is maintained within the same order. Another hypothesis is that gene diversity is driven by environmental pressures. Our dataset includes copepods from different habitats: the sub-Arctic Ocean, the Southern Ocean (Antarctic waters), and the Mediterranean Sea. Despite the obvious environmental differences (e.g., temperature, salinity, chlorophyll a) between these environments, we found similar relative contributions of the different genes in the species inhabiting these water masses. However, the abundances of some genes were different between the Mediterranean Sea and the Subarctic and Antarctic copepod species (Table 1). *R. gigas*, the only individual from Antarctic waters, showed a lower percentage of genes belonging to the *ABC-cassette* family, *ALDH*, *CYP450*, and *SULT* groups. Some differences were also found in copepods from the Subarctic in terms of a lower percentage of *GST*, *SOD*, and *UGT* genes (Table 1). Overall, the highest multiplicity of genes in copepods from the Mediterranean basin, compared with copepods living in polar areas, could reflect an adaptive evolutionary strategy of the species to cope with living in more variable environmental conditions [31]. Copepods inhabiting Mediterranean waters might experience a wide range of natural toxins because of the high phytoplankton diversity, as well as human pollutants with variable levels of toxicity. Thus, having more genes can represent a fitness advantage to metabolize the variety of xenobiotics and natural toxins encountered in the environment. Considering that the Arctic ecosystems are considered at risk due to high levels of chemical contamination, in terms of Persistent Organic Pollutants (POPs), mercury, and other new Chemicals of Emerging Concern (CECs) [22,32,33,34], efforts should be made in the future to assess whether cold-water copepods might be more vulnerable to the detrimental effects associated with these chemicals than their temperate counterparts.

In our study, we found a high consistency in the expression of defensome genes in copepods exposed to toxic phytoplankton species in laboratory and field conditions. Different *GST* (sigma and theta) and *SOD* (Cu-Zn and Mn) gene isoforms were significantly downregulated in *C. helgolandicus* and *T. stylifera* copepods feeding on oxylipin-producing diatoms. This agrees with what was shown by Lauritano et al. [35] and Asai et al. [36] for *C. helgolandicus* feeding on the oxylipin-producing diatom *Skeletonema marinoi* in laboratory experiments. The reduced expression of *GST* and *SOD* genes after exposure to harmful oxylipins could be a direct inhibitory effect of the algal toxin of the copepod defensive system; alternatively, it is possible that the toxins ingested were not sufficient to induce a detoxification response in the copepods, or that the copepods were compensating with the upregulation of other defensive genes or pathways. Notwithstandingly, the downregulation of stress genes has been reported in other copepods or arthropods exposed to biotoxins and anthropogenic pollutants. For example, *GST* sigma and theta were downregulated in *Tigriopus japonicus* feeding on the toxic dinoflagellate *Gymnodinium catenatum* (Graham, 1943) [37] and in *T. japonicus* exposed to organic pollutants [38]; also, a lower expression was observed in the Chinese mitten crab *Eriocheir sinensis* (Milne Edwards, 1853) exposed to cadmium [39] and in the insect *Chironomus riparius* (Meigen, 1804) exposed to phthalates [40]. Recently, a significant downregulation of *Cu-ZnSOD* and *GST* sigma genes has been reported in *A. clausi* and *Acartia tonsa* (Dana, 1849–1852) exposed to NiCl2 and in *A. clausi* exposed to Ni nanoparticles, at concentrations that reduced the copepod’s fecundity but not its survival [41].

In our study, we also found that the *GST1* delta isoform was, on the contrary, upregulated in *C. helgolandicus* feeding on *S. marinoi*, as well as in *C. finmarchicus* fed the harmful dinoflagellate *A. fundyense* and in *A. clausi* exposed to highly polluted industrial sediments. It is possible that the *GST1* gene can actively participate in the detoxification process of biotoxins and anthropogenic chemicals (PAHs and HMs) in different copepod species. Further studies are needed to verify whether the *GST1* gene is part of a “common gene core” of the chemical defensome of copepods. *A. clausi* exposed to the polluted sediment also showed an upregulation of genes belonging to other defensome gene classes: *ABC* (transporter), *CYP450* (phase I), *GSTs* (phase II), and *SOD* (antioxidant). This agrees with previous observations showing an upregulation of GST sigma, *Cu-ZnSOD*, and *CAT* in *A. clausi* [41]. It is possible that the concerted activation of all these defensive genes has contributed to the detoxification of the chemical mixture, thus resulting in the survival of the exposed copepods [42]. A similar upregulation of detoxification *CYP450* and *GST* genes has been reported in other marine invertebrates, such as the polychaete *Perinereis nuntia* (Lamarck, 1818) exposed to PAHs [43], the greentail prawn *Metapenaeus bennettae* (Racek and Dall, 1965) [44], and the amphipod *Melita plumulosa* (Zeidler, 1989) [45] exposed to crude oil and in the copepod *E. affinis* exposed to PHAs [46].

## 4. Materials and Methods

### 4.1. In Silico Mining for Defensive Genes

Eleven transcriptomic and one genomic resource were surveyed for defensive genes, including efflux transporters (*ABC* proteins), phase I enzymes (*CYP450*, *FMO*, *ALDH*), phase II enzymes (*GST*, *SULT*, *UGT*, *AKR*), and antioxidants (*SOD*). This subset of defensome gene classes was chosen to enable comparisons with previous studies from *N. vectensis* [1], *S. purpuratus* [2], *E. affinis*, and *C. finmarchicus* [22]. Of the 11 copepod transcriptomes surveyed, the majority belong to the Calanoida order, with one member from the Siphonostomatoid order (*Lepeophtheirus salmonis*) and one from the Harpacticoid order (*T. calfornicus*) (Figure 1). Among the calanoid copepods, the Calanidae family was the most represented, including *C. helgolandicus*, *Neocalanus cristatus*, *N. flemingeri*, and *N. plumchrus*, followed by the Temoridae family with *Eurytemora affinis* and *Temora stylifera* and a single member for the Acartiidae (*Acartia clausi*), Eucalanidae (*Eucalanus bungii*), Metridinidae (*Metridia pacifica*), Pontellidae (*Labidocera madurae*), and Rhincalanidae (*Rhincalanus gigas*) families. More than half of the mined transcriptomes (6/11) were from adults, including 4 from females, 1 male (*N. plumchrus*), and 1 not specified (Figure 1). Three transcriptomes were generated from the pre-adult CV stage and two from a pool of mixed developmental stages (*L. madurae*) (Figure 1). In terms of geographical distribution, our study includes transcriptomes generated from individuals collected from the Gulf of Naples (*A. clausi*, *C. helgolandicus*, and *T. stylifera*), the Gulf of Alaska (*N. cristatus*, *N. flemingeri*, *N. plumchrus*, *E. bungii*, and *M. pacifica*), Kāne`ohe Bay (Hawai’i; *L. madurae*), and the South Shetland Trench (Southern Ocean; *R. gigas*); lastly, two transcriptomes originated from individuals reared in laboratory conditions, namely *T. calfornicus* (strain San Diego, Pacific Ocean) and *Lepeophtheirus salmonis* (strain Norway) (Figure 1).

All transcriptomes were generated from RNASeq reads (paired-end) sequenced on Illumina platforms (San Diego, CA, USA) and *de novo* assembled using Trinity software, except for *L. salmonis*, whose reads were sequenced on Illumina and PacBio and assembled with the software LorDEC (v. 0.9) (Table S1). For 9 (out of 11) transcriptomes, functional annotations were generated through blast-based comparisons against the Nr, SwissProt, and RefSeq databases (Table S1). For the identification of the defensive genes, the annotated files, publicly available for the majority of the sources (Table S1), were searched by name-generating an initial list of genes (*Tigriopus calfornicus*) or transcripts encoding proteins for the following classes: *ABC* proteins, phase I enzymes (*CYP450*, *FMO*, *ALDH*), phase II enzymes (*GST*, *SULT*, *UGT*, *AKR*), and antioxidant (*SOD*). Then, to account for transcriptomic differences associated with coverage and/or the fragmentation, a Transdecoder analysis was performed on the 11 transcriptomes. Briefly, transcriptomes were downloaded from the NCBI transcriptome shotgun assembly (TSA) database. A Transdecoder analysis (v. 5.5.0; setting: report only the single best open reading frames [ORFs]) was performed for all transcriptomes (same settings) to determine the encoding open reading frames (ORFs). As a final step, the list of transcripts identified by name in the annotation file (step 1) was searched in the list of transcripts generating ORFs (Transdecoder). Thus, for each copepod, the final list of defensive genes included only annotated transcripts encoding opening reading frames (ORFs).

### 4.2. Relative Expression of Chemical Defensome Genes When Exposed to Stressors

To gain further details, the relative expression of defensome-related transcripts was investigated for the copepods *C. helgolandicus*, *C. finmarchicus*, *T. stylifera*, and *A. clausi* exposed to environmental stressors using existing RNASeq data [35,36,41,47]. The *C. finmarchicus* dataset included gene expression data from females collected from the Gulf of Maine and exposed to the saxitoxin-producing dinoflagellate *Alexandrium fundyense* [23]. Briefly, field-collected adult females were laboratory-incubated over a week with a low dose (LD: 50 cell/mL) and high dose (HD: 200 cell/mL) of the toxic dinoflagellate and with the non-toxic cryptophyte *Rhodomonas baltica* (8 × 10^3^ cells/mL) as the control diet. At two and five days, three biological replicates (15 females each) were harvested, extracted for total RNA, and processed for RNASeq [23]. The data presented here are shown only for the 2 days. *C. helgolandicus* RNASeq data was generated from females collected in the Gulf of Naples and laboratory-incubated for five days with the oxylipin-producing toxic diatom *Skeletonema marinoi* (SKE) (45 × 10^4^ cells/mL) and the dinoflagellate control diet *Prorocentrum minimum* (PRO) (5 × 10^3^ cells/mL) [36]. After five days, three biological replicates (10 females each) were harvested, extracted for total RNA, and processed for RNASeq. The *T. stylifera* dataset included females collected in the Gulf of Naples during two consecutive weeks (May 2017) with differences in the natural phytoplankton oxylipin content. Briefly, females from the first week were exposed to a higher content of phycotoxins compared with females from the second week (test: 23rd and control: 30th). At each time point, females were immediately harvested (three replicates of 10 females each), extracted for total RNA, and processed for RNASeq [48]. Lastly, we also examined the expression of defensive genes in *A. clausi* exposed to elutriates of polluted sediments (containing high concentrations of polycyclic aromatic hydrocarbons, PAHs, and heavy metals) from an industrial area in the Southern Tyrrhenian Sea (Bagnoli-Coroglio) [40]. Briefly, *A. clausi* females collected from the Gulf of Naples were incubated with the elutriate (E56) and filtered seawater (control) and fed the cryptophyte *Rhinomonas reticulata* (3 × 10^4^ cells/mL), harvested after 4 days (9–10 females in each replicate), extracted for total RNA, and processed for RNASeq [41]. For each dataset, expression levels were obtained using the mapping data of the RNASeq libraries against their species-specific reference transcriptome.

### 4.3. Statistical Analyses

Counts were normalized by length using the reads per kilobase per million mapped reads (RPKM) method [49]; statistical tests (multiple unpaired *t*-tests [*p* < 0.05]) with the *p*-value corrected for multiple comparisons using the Holm–Sidàk method were performed to identify transcripts with significant differences in expression between control and treatment (GraphPad Software v. 9, San Diego, CA, USA).

## 5. Conclusions

This study provides the first in-depth investigation of the defensome in marine copepods reporting transcripts encoding proteins divided into transporters and phase I and phase II detoxification enzymes. The chemical defensome is essential for the detoxification and subsequent clearance of xenobiotic compounds, and the composition of the defensome can determine the toxicological responses to many chemicals. The genes presented here can be used as biomarkers for future studies investigating ecosystem health and organism–environment interactions and can be tested not only in other copepods but also other zooplankters. The use of these biomarkers to assess the “health status” of a marine organism is important for understanding environmental adaptations on a broader scale to assess changes and make predictions at population and community levels. A comparative analysis of gene expression between different copepods and stressors highlights that *GSTs* and *SOD* are good targets of biotic and abiotic chemical stress in copepods, whereas *ABC* and *CYP450* are likely related to very toxic chemical exposure (anthropogenic chemicals, such as PAHs and HMs, or PSP toxins). However, deeper investigations are necessary to enhance the mechanistic understanding of exposure–effect relationships, which may also be useful for risk assessments induced by pollutant mixtures and the evaluation of ecological effects associated with harmful algal blooms.

## Figures and Tables

**Table 1:** Summary of transcripts encoding enzymes identified in copepods living in the Mediterranean basin, Subarctic, and Antarctic habitats. Transcripts have been classified into four major classes (transporters, phase I and phase II detoxification, and antioxidant enzymes) (see text for more details). The number of transcripts encoding proteins for each habitat refers to the average of the different copepods included in that category.

	Mediterranean (n = 6) ^1^	Subarctic (n = 5)	Antarctic (n = 1)
Transporters			
ABC	37	28	20
Phase I detoxification enzymes			
ALDH	9	8	4
CYP	36	26	22
FMO	2	1	2
Phase II detoxification enzymes			
AKR	4	3	2
GST	28	16	24
SULT	8	8	3
UGT	11	5	10
Antioxidant enzyme			
SOD	12	5	12

^1^ Number of copepods included in the category.

## Data Availability

The original data (transcriptomes, annotation files) surveyed in this study are openly available in NCBI as listed in Table S1. For the annotation files not available, further inquiries can be directed to the authors of the original study.

## Supplementary Materials

The following supporting information can be downloaded at: https://www.mdpi.com/article/10.3390/ijms26041546/s1 [50,51].
