# Supplementary material for: The Chemical Defensome: A Survey of Environmental Sensing and Response Genes in Copepods
Source: Int J Mol Sci. 2025 Feb 12;26(4):1546. doi: 10.3390/ijms26041546 (PMC11855160; doi:10.3390/ijms26041546)
Supplement: Supplementary file 1 [file ijms-26-01546-s001.zip › ijms-3409652-supplementary.pdf]

Species name	NCBI Accession n.	Sequencing platform	Source[1]	Assembly software (version, settings)[2]	Functional annotation	Ref annotation
Acartia clausi	PRJNA973259 GKQN00000000	NextSeq 75PE	T	Trinity (v. 2.0.6)[2]	NCBI NR 10^{-3}	Authors personal contribution
Calanus helgolandicus	PRJNA640515 GJFL00000000	HiSeq2000 100PE	T	Trinity (v. 2013-02-25)[3]	NCBI Swiss-Prot protein and NR 10^{-6}	Authors personal contribution
Temora stylifera	PRJNA632714 GJGX00000000	HiSeq2000 100PE	T	Trinity (np)	NCBI NR 10^{-3}	Authors personal contribution
Neocalanus cristatus	PRJNA662858 GJRH00000000	NextSeq500 150PE	T	Trinity (v. 2.4.0)[2]	NCBI Swiss-Prot protein 10^{-5}	[50]
Neocalanus flemingeri	PRJNA496596 GHLB01037025	NextSeq500 150PE	T	Trinity (v. 2.4.0)[2]	NCBI Swiss-Prot protein 10^{-5}	[50]
Neocalanus plumchrus	PRJNA662858 GJRU00000000	NextSeq500 150PE	T	Trinity (v. 2.0.6)[2]	NCBI Swiss-Prot protein 10^{-5}	[50]
Eucalanus bungii	PRJNA662858 GJRG00000000	NextSeq500 150PE	T	Trinity (v. 2.4.0)[2]	NCBI Swiss-Prot protein 10^{-5}	[50]
Metrdia paciifiica	PRJNA662858 GJAO00000000	NextSeq500 150PE	T	Trinity (v. 2.4.0)[2]	NCBI Swiss-Prot protein 10^{-5}	[50]

Tigriopus calfornicus	PRJNA237968<br>VCGU00000000	Illumina/PacBio	G	HiRise v. 2017-07 (np)	NCBI RefSeq (Evalue np)	https://ncbi.nlm.nih.gov/search/all/?term=GCF_007210705
Labidocera madurae	PRJNA324849<br>GFWO01000000	NextSeq500 150PE	T	Trinity (v. 2.0.6)[2]	NCBI Swiss-Prot protein 10^{-3}	https://demo.bco-dmo.org/project/542182
Rhincalanus gigas	PRJNA639356	NextSeq500 75PE	T	Trinity (v. 2.0.6)[2]	NCBI Swiss-Prot protein 10^{-3}	Authors personal contribution
Lepeophtheirus salmonis	PRJNA937046<br>GKKU00000000	Illumina/PacBio	T	LoRDEC 0.9[4]	ENSEMBL, RefSeq (Omicsbox software E 10^{-15})	[51]

[1]T=Transcriptome; G=Genome

[2] Trinity settings: --seqType fq --CPU 32 --max_memory 300G -- min_contig_length 300 -- normalize_max_read_cov 50

[3] Trinity settings: --seqType fa --JM 200G --inchworm_cpu 20 --bflyHeapSpaceInit 20G --bflyHeapSpaceMax 200G --bflyCalculateCPU --CPU 20 &.

[4] Settings: k-mer size 21 and solidity threshold
